# The possible role of seasonality and hs-CRP in the evaluation of suicidality risk

**DOI:** 10.1192/j.eurpsy.2025.2030

**Published:** 2025-08-26

**Authors:** G. Longo, L. Cavallo, L. Orsolini, U. Volpe

**Affiliations:** 1 Department of Neurosciences/Department of Experimental and Clinical Neurosciences (DIMSC), Polytechnic University of Marche, Unit of Clinical Psychiatry, Ancona, Italy

## Abstract

**Introduction:**

Suicidality is a critical concern in individuals with affective disorders, and environmental and biological factors may influence its risk. In recent years, seasonal variations and systemic inflammation, as indicated by high-sensitivity C-reactive protein (hsCRP), have garnered interest as potential contributors.

**Objectives:**

To assess the risk of suicidality in affective disorders among seasonality correlated with hsCRP.

**Methods:**

A naturalistic, observational, cross-sectional study was carried out by retrospectively recruiting 353 adult inpatients affected by severe mental illness (SMI) consecutively hospitalized in the Psychiatry Clinic of the Ospedali Riuniti of Ancona, Italy. Patients affected by inflammatory pathology, alcohol/substance use disorders or treated by anti-inflammatory/immunosuppressive therapy were excluded. Only patients suffering from mood disorders were considered for this analysis (n=246). We administered a checklist for socio-demographic and clinical features (diagnosis, age of onset, disease duration, number of episodes, number of episodes per year, suicidal attempts and comorbidities). Subscale 5 of the Mini International Neuropsychiatric Interview (MINI-5-s) was administered to all the patients involved to assess suicidality risk. To normalize hs-CRP, a logarithmic transformation was performed (log-hsCRP). At the same time, season were codified as dummy variables. T-test for independent groups and multivariate linear regression were conducted.

**Results:**

47.2% (n=116) of the sample were male. The mean value of hsCRP was 5.7mg/L (SD=15.8). The mean score of MINI-5-s total score was 13.3 (SD=11.6). Patients admitted to our psychiatric ward in meteorological (p=0.013) and astronomical (p=0.049) autumn had a lower log-hsCRP compared to other seasons. A multivariate linear regression was observed between MINI-5-s total score (R² =0.74, F=9.639, Durbin-Watson=1.915, p<0.001) and hsCRP (B=0.94, p=0.041) and the meteorological autumn (B=-7.436; p<0.001).

**Conclusions:**

This study highlights a significant association between seasonality, systemic inflammation (as measured by hsCRP), and suicidality risk in patients with mood disorders. Our results focus on the importance of considering biological and environmental factors in assessing and managing suicidality risk in affective disorders. Further studies are essential to link seasonal and inflammatory mechanisms.

**Disclosure of Interest:**

None Declared

